# Dietary *Spirulina platensis* phycocyanin enhances growth performance, antioxidant status, and immune-related gene expression in Japanese quails (*Coturnix japonica*)

**DOI:** 10.1038/s41598-026-45365-9

**Published:** 2026-04-19

**Authors:** Basma M. Hendam, Menna H. E. Morsy, Rabab Mohamed Aljarari, Safaa Abdullah Alowaidi, Moaheda E.H. Eissa, El-Sayed Hemdan Eissa, Yasmin M. Abd El-Aziz

**Affiliations:** 1 Department of Animal Wealth Development, Faculty of Veterinary Medicine, Mansoura University, Mansoura, 35516 Egypt; 2 Zoology Department, Faculty of Science, Arish University, El-Arish, 45511 Egypt; 3 Department of Biological Sciences, College of Science, University of Jeddah, P.O. Box 80327, Jeddah, 21589 Saudi Arabia; 4 Department of Biology, Faculty of Sciences, King Abdulaziz University, Jeddah, Saudi Arabia; 5 Biotechnology Department, Fish Farming and Technology Institute, Suez Canal University, Ismailia 41522, Egypt; 6 Fish Research Centre, Faculty of Environmental Agricultural Sciences, Arish University, El Arish, Egypt; 7 Department of Zoology, Faculty of Science, Port Said University, Port Said, 42526 Egypt

**Keywords:** Quails, Phycocyanin, Gene Expression, Growth, Histology, Biochemistry, Physiology, Zoology

## Abstract

The study aimed to evaluate the effects of dietary phycocyanin extracted from *Spirulina Platensis* (PSP) supplementation on the expression of antioxidant and immune associated genes, growth performance related parameters, blood biochemical components, and hematological profiles in Japanese quails (*Coturnix japonica*). A total of 320 male Japanese quails, 7 days old, were randomly assigned to four experimental groups, each consisting of four replicates with 20 quails per replicate (80 quails per group). The quails were fed basal diets supplemented with PSP at concentrations of 0 (PSP0), 1 (PSP1), 2 (PSP2), and 3 (PSP3) g/kg of diet. The results showed that quails fed diets supplemented with 3 g of PSP /kg diet had significantly better growth performance and feed efficiency compared to the other groups (*P* < 0.001). Increasing dietary PSP levels resulted in a significant, dose-dependent improvement in hematological parameters (*P* < 0.001). Dietary supplementation with PSP (1, 2–3 g/kg diet) resulted in a significant dose-dependent increase in total protein (TP), triglycerides (TG), globulins, and lysosomal activity (*P* < 0.001). Conversely, serum levels of alanine aminotransferase (ALT), creatinine, total cholesterol (TC), and urea exhibited a significant, dose-dependent decrease with increasing PSP supplementation (*P* < 0.001). Additionally, including PSP in the diets of Japanese quails led to significant improvements in small intestinal and liver morphology. Dietary supplementation with PSP significantly upregulated the mRNA expression of antioxidant defense genes (Catalase (*CAT*) and glutathione peroxidase (*GPX*)). However, the mRNA expression of pro-inflammatory cytokine genes, specifically interleukin 8 (*IL-8*) and interleukin 1 beta (*IL-1β*), exhibited a dose-dependent decrease with elevated dietary phycocyanin extracted from PSP concentrations. In conclusion, dietary supplementation with PSP promotes growth performance and feed efficiency, enhances antioxidant defense, upregulates growth-related gene expression, and improves organ histomorphology.

## Introduction

As dependable sources of sustenance, meat and eggs provide a rich source of protein for human diets. The industry of Japanese quail (*Coturnix japonica*) has increased recently due to several benefits, such as low-fat meat and eggs, high egg production, and little space requirements^1^. Feed consumption has increased as a result of the chicken industry’s growth, and diets are being manipulated to maximize efficiency and conversion rates. Utilizing materials that may effectively support these birds’ growth and well-being will therefore be advantageous^2^. In the poultry industry, the extensive usage of antibiotics creates a selective environment that encourages the growth of bacteria resistant to antibiotics^3,4^. Direct contact with diseased animals or consumption of tainted livestock and poultry feed products can spread these germs, which can infect humans and animals^5–7^.

The use of antibiotics in feed production has led to a decrease in the diversity of normal gut microbiota, antibiotic residues and resistance in feed^8,9^. Because these substances lead pathogenic microbes acquired in human microbiota to become resistant to these antibiotic families, the incidence of antibiotic residues in chicken products, such as meat or eggs, is harmful to human well-being^10^. Numerous reports have documented the beneficial effects of natural substances (such as phytobiotics, prebiotics and probiotics) on immunity, growth, and nutrients digestibility^11,12^. Phytobiotic algae have been notified to have valuable consequences as a dietary supplement for humans. Algae may boost immunity and reduce inflammation, according to several human studies^13–17^.

Algae might be a unique protein source for poultry diets and a good alternative to other proteins, claims^18^. Spirulina is a blue-green algae commonly used in feed supplements^19^. Carbohydrates account for 15–25%, proteins for 50–70%, and minerals, fatty acids, vitamins, carotenes, phenolic acids, chlorophyll, gamma-linoleic acid, and phycobiliprotein pigments make up about 18% of the composition^18^. Investigations by Altmann et al.^20^ suggested that *Spirulina platensis* could be a possible source of protein for poultry diets. In recent years, many other pharmacological traits of Spirulina have been identified. Additionally, studies suggest that Spirulina may improve intestinal *lactobacilli*, minimize nephrotoxicity incidence from heavy metals and medications, provide protection from radiation, and have therapeutic advantages^19,21^.

Phycocyanin is one of spirulina’s active ingredients. Spirulina’s cytoplasmic membrane contains the water-soluble blue photosynthetic pigment known as phycocyanin. Phycocyanin exhibits anti-inflammatory, anti-tumorigenic, antioxidant, and radical scavenging qualities because of its alkyl, hydroxyl, and aromatic groups^22^. Spirulina possesses antioxidant properties that have been shown to prevent organ damage and inhibit the growth of cancer^23^. Spirulina has been used globally to enhance the color of egg yolks and poultry flesh. It has also been added to the diets of high-quality broilers to improve their overall growth, reproduction, and immune function^24^. Supplementing hens with spirulina is thought to enhance their growth rate, survival rate, feed consumption, and carcass quality^25^. This aligns with broader research suggesting that microalgae can be a valuable addition to poultry feed^24^. Adding a high dose of *Spirulina platensis* to the diet of Japanese quails can improve their growth performance and immune responses^26^. More broadly, research suggests that microalgae can contribute to the healthy growth and feed efficiency of broilers. According to Ahmed et al.^27^ dried spirulina is a valuable new animal feed, containing roughly 60% protein. Its antibacterial properties and ability to strengthen both humoral and cell-mediated immunity in chickens promote disease resistance, resulting in improved survival and growth rates^28^. Based on previous studies, using spirulina in poultry diets has improved growth, health, and immune function due to its content of several biological molecules, such as phycocyanin^29,30^. We hypothesized that adding phycocyanin to the diet of Japanese quails (*Coturnix japonica*) could enhance growth and health by regulating tissue integrity, antioxidant status, and inflammatory pathways. Therefore, this study aimed to investigate the effect of phycocyanin extracted from *Spirulina Platensis* on antioxidant/pro-inflammatory cytokines genes pathways, growth attributes, hematological indices, blood metabolites and histology of Japanese quails (*Coturnix japonica*).

## Materials and methods

### Ethical approval

All procedures, protocols, and animal handling have been reviewed and approved by the Mansoura University Committee. The protocol of the study was approved by the Research Ethics Committee, Faculty of Veterinary Medicine, Mansoura University with code;  MU-ACUC (VM.R.25.10.248). We confirm that all methods were performed in accordance with the relevant guidelines and regulations.

### Birds and experimental diets

All birds in this experiment were obtained from a private farm. Three hundred and twenty 7-day-old healthy male Japanese quail birds (*Coturnix japonica*), with an average weight of 33.90 g were randomly distributed into four treatment groups with 80 birds per group and four replicates (*n* = 20 birds per cage) in each group using a completely randomized design.

Experimental birds were divided into four groups. The control group received a basal diet, while the PSP1, PSP2, and PSP3 groups were fed diets containing 1, 2, and 3 g/kg of phycocyanin extracted from *Spirulina platensis* (PSP), respectively, according to El-Abd et al.^31^. The duration of the study was 35 days.

The basal diet was prepared to satisfy the nutrient requirements of Japanese quails according to NRC^32^(Table 1). All feed ingredients were based on corn and soybean meal to meet the nutritional requirements proposed by Rostagno et al.^33^.

**Table 1:** The basal diet and its calculated composition used in this experiment for Japanese quails (*Coturnix japonica*).

Ingredients	Ingredients %			
	Control (PSP0)	PSP1	PSP2	PSP3
Corn	48.00	47.90	47.80	47.70
PSP	0.00	0.10	0.20	0.30
Rice polishing	9.00	9.00	9.00	9.00
Rice broken	8.00	8.00	8.00	8.00
Soybean meal	19.00	19.00	19.00	19.00
Sunflower meal	4.00	4.00	4.00	4.00
Canola meal	7.00	7.00	7.00	7.00
Feather meal	2.00	2.00	2.00	2.00
Fish meal	1.00	1.00	1.00	1.00
Molasses	1.00	1.00	1.00	1.00
Premix*	1.00	1.00	1.00	1.00
Chemical composition (%)				
Crude Protein %	20.88	20.86	20.86	20.88
Metabolizable energy Kcal/Kg	2883	2890	2879	2883
Lysine	1.34	1.34	1.35	1.32
Methionine	0.35	0.36	0.35	0.35

*Premix: Each 2.5 kg consists of vit. A 12,000 IU; vit. D3, 2000 IU; vit. E, 10 g; vitk3, 2 g; vitB1, 1000 mg; vitB2, 4 g; vitB6, 1.5 g; vitB12, 10 mg; pantothenic acid, 10 g; niacin, 20 g; folic acid, 1000 mg; biotin,50 mg; choline chloride, 500 g; Fe, 30 g; Mn, 40 g; Cu, 3 g; Co, 200 mg; Si, 100 mg and Zn, 45 g.

Japanese quails were accommodated in conventional-type bird cage (90 × 40 × 40 cm^3^) with free access of water and feed throughout the study. The ambient temperature gradually diminished from 33 °C to 25 °C on day 22 and then resided persistent. On day one, the ambient temperature was first set at 33 °C and slowly diminished by 2–3 °C per week until reaching 24–25 °C. The relative humidity was maintained at 45–55% from days 1 to 14, after which it was increased to 65–70% for the remainder of the study. The lighting program consisted of a 23 L:1D photoperiod (23 h of light and 1 h of darkness) with a light intensity of 25 lx. The stocking density was set at 20 birds per square meter for each cage.

### Growth performance

Individual live body weights (LBW) of quails were measured after 35 days of treatment using an electronic digital balance. Body weight gain (BWG) was subsequently calculated for each experimental period. Feed intake (FI) was recorded on a replicate basis throughout the experimental periods to determine the feed conversion ratio (FCR), calculated as grams of feed consumed per gram of weight gained.

### Blood sampling and hematological assays

At 35 days of age, 10 birds per group were fasted for 12 h. Following the fast, birds were randomly selected and slaughtered by severing the neck and jugular veins for blood sample collection, in accordance with the Islamic method described by Mylostyvyi et al.^34^. Anesthesia was given as Ketamine 20 mg per kg of body weight and Diazepam (at 2 mg/kg b.wt. IM). Quails were humanely slaughtered via ventral neck incision, severing the jugular veins and carotid arteries in accordance with AVMA guidelines. This procedure was performed by competent technicians to ensure rapid loss of consciousness and effective exsanguination. The samples were divided into two equal portions. The first was collected in EDTA tubes for hematological analysis. The second portion was collected and immediately centrifuged at 3,000 rpm for 15 min to separate the plasma. The isolated plasma was then stored at -20 °C for later measurement of biochemical characteristics. We measured the following blood hematological parameters: white blood cells (WBCs), red blood cells (RBCs), hemoglobin (Hb), and packed cell volume (PCV). The white blood cell differential count included Eosinophils, Lymphocytes, Monocytes, and Neutrophils. We also determined the erythrocyte indices, which included mean corpuscular volume (MCV), mean corpuscular hemoglobin (MCH), and mean corpuscular hemoglobin concentration (MCHC). The previously mentioned parameters were analyzed using a Prima Fully-Auto Hematology Analyzer (PT. Prima Alkesindo Nusantara, Jakarta, Indonesia) in accordance with the manufacturer’s protocol^35^. During the slaughtering process, we collected tissue samples from the experimental birds. Liver tissue was immediately stored in liquid nitrogen for subsequent RNA extraction and real-time PCR analysis. Additionally, intestine and liver tissue samples were collected for histopathological analysis.

### Blood metabolic parameters

Five plasma samples from each group were used to investigate changes in blood metabolic parameters in response to the PSP dietary addition. The plasma levels of total protein and albumin were assessed using specific Biodiagnostic ELISA kits, following the manufacturer’s instructions (Biodiagnostic, Giza, Egypt). To determine the globulin concentration, we subtracted the albumin value from the total protein value in the serum. Serum samples were investigated for concentrations of total protein (TP), total cholesterol (TC), albumin (ABU), triglycerides (TG), creatinine, and urea. The globulin was calculated by the difference between TP and ALBU. Additionally, the levels of aspartate (AST) and alanine (ALT) aminotransferase were verified spectrophotometrically by commercial kits from Biodiagnostic Company (Giza, Egypt). Lysosome activity was determined in accordance with the method outlined by Eldiasty et al.^36^.

### Histological study

Specimens from the intestine and liver of quails were collected and directly fixed in 10% buffered neutral formalin liquid for 24 h, dehydrated in gradually ascending ethanol (70%, 80%, 95%, 95%, and 100%), cleared in xylene, and embedded in paraffin. Paraffin sections of 5 μm thickness were sliced using a microtome (Leica RM 2155, England). The sections were then stained with hematoxylin and eosin stains and examined microscopically^14,15^.

Masson’s trichrome stain was used for the liver sections, and periodic acid-Schiff (PAS) stain was used for the intestine sections. The PAS histochemical reactivity area percentage was calculated using Image J software, and the average number of goblet cells was determined for each group.

We measured the intestinal villus width, villus length, and absorption surface area. For each fish, we evaluated these variables on 50 well-aligned villi from each section of all intestinal segments and then calculated the average. The villus length (VL) was measured from the tip to the base, while the villus width (VW) was assessed at the midpoint. We analyzed the tissue samples using a light microscope equipped with an HD microscopic camera and image analysis software (Leica Microsystems, Germany). The variables were then processed using picture investigation software for statistical analysis. The absorption surface area (ASA) was calculated using the following formula, as described by Rehman et al.^37^.

$${\text{ASA }}\left( {{\mathrm{mm}}^{{\mathrm{2}}} } \right) = {\mathrm{Villus}}~{\text{height }}\left( {\upmu {\mathrm{m}}} \right) \times {\mathrm{Villus}}~{\text{width }}\left( {\upmu {\mathrm{m}}} \right).$$

### Gene expression

We extracted total RNA from 50 mg of hepatic tissue from each experimental group using an RNA purification kit (Thermo Fisher Scientific, USA), following the manufacturer’s protocol. We assessed the purity of the isolated RNA using a Nanodrop Lite spectrophotometer by measuring the O.D. 260 nm/O.D. 280 nm ratio (Thermo Scientific, USA).

We synthesized complementary DNAs (cDNAs) from 1 µg of RNA using the SuperScript™ III First-Strand Synthesis System and Oligo-dT primers (Invitrogen, USA), according to the manufacturer’s guidelines. The cDNA samples were then stored at -20 °C for future use. We quantified the messenger RNA (mRNA) expression of several target genes using qPCR with a SensiFast SYBR Lo-Rox kit (Bioline, London, UK). The genes analyzed included those related to antioxidant capacity (Catalase (*CAT*) and glutathione peroxidase (*GPX*) and pro-inflammatory cytokines (interleukin 8 (*IL-8*) and interleukin 1 beta (*IL-1β*) (Table 2). We used the following thermal cycling conditions for the reaction: initial denaturation at 95 °C for 10 min, followed by 40 cycles of 95 °C for 15 s and 60 °C for 30 min, with a final step at 85 °C for 5 min. We normalized the transcription levels to the GAPDH gene and quantified gene expression using the 2^−ΔΔCT^ method^38^. The accession numbers of confirmed sequences specific for Coturnix japonica primers that were used in this study were not available on NCBI Primer-BLAST, so predicted accession numbers were used instead.

**Table 2:** Sequence of forward and reverse primers used for q-PCR analysis.

Gene	Primer sequence	Genbank accessation no.	Product size
*CAT*	F: CCTGACTATGGTGCGCGTAT R: CAGACACACGAGAAGTGGCT	XM_015863594.1	106 bp
*GPX*	F: TTGTAAACATCAGGGGCAAA R: TGGGCCAAGATCTTTCTGTAA	XM_015870585.2	140
*IL-8*	F: CTGAGGTGCCAGTGCATTAG R: AGCACACCTCTCTTCCATCC	XM_015861822.2	139 bp
*IL-1β*	F: CTTCCTCCAGCCAGAAAGT R: CAGCTTGTAGCCCTTGAT	XM_015882931.2	116 bp
*GAPDH*	F: TCTCTGTTGTTGACCTGACCTG R: ATGGCTGTCACCATTGAAGTC	XM_015873412.2	155 bp

Catalase (CAT) and glutathione peroxidase (GPX), interleukin 8 (IL-8) and interleukin 1 beta (IL-1β).

### Statistical analysis

All data were initially organized in Microsoft Excel version 16 (Microsoft Corporation, Redmond, WA) and assessed for normality and homogeneity with the Shapiro-Wilk test. We performed a one-way ANOVA using SPSS version 26.0 (IBM Co., Chicago, IL), followed by Duncan’s multiple-range test for post hoc comparisons. Statistical significance was set at a p-value < 0.05. Results are expressed as the mean ± standard deviation, and all figures were generated using GraphPad Prism 9.0 (GraphPad, University of California San Diego).

The statistical model was used for the analysis of variation to evaluate the influence of different levels of raffinose as follows:

$$\:\mathrm{Y}\mathrm{i}\mathrm{j}=\mathrm{U}+\mathrm{T}\mathrm{i}+\mathrm{e}\mathrm{i}\mathrm{j}$$

where Y_ij_, observation; µ, observed mean; T_i_, effect of treatments; e_ij_, experimental random error.

## Results

### Growth performance

All supplemented groups had a greater final body weight (FBW) and body weight gain (BWG) than the control groups. Birds fed various levels of PSP (0, 1, 2, 3 g/kg diet) showed a dose-dependent increase in FBW (*P* < 0.001) and BWG (*P* < 0.01) (Table 3). Feed intake (FI) did not differ among all PSP supplemented groups and the control diets (*P* > 0.05). Adding a dietary supplement of PSP significantly reduced the feed conversion ratio (FCR), indicating improved feed efficiency (*P* < 0.05). Specific growth rate (SGR) was significantly improved in all PSP-added groups (*P* < 0.05). Average daily gain (ADG) was significantly improved by the addition of PSP (*P* < 0.01). Overall, supplementing the diet of growing Japanese quails with phycocyanin significantly improved their growth indices and feed efficiency.

**Table 3:** The effects of dietary inclusion of phycocyanin extracted from *Spirulina Platensis* (PSP, 0, 1, 2, and 3 g/kg diet) on the growth indices of growing Japanese quails.

Item	Dietary addition groups				*P* values
	PSP0	PSP1	PSP2	PSP23	
IBW, g	33.90 ± 0.14^a^	33.90 ± 0.14^a^	33.85 ± 0.15^a^	33.83 ± 0.18^a^	0.479
FBW, g	195.73 ± 0.94^d^	206.38 ± 1.04^c^	211.23 ± 0.79^b^	214.75 ± 1.12^a^	< 0.001
BWG, g	161.83 ± 0.84^d^	172.48 ± 0.95^c^	177.38 ± 0.83^b^	180.93 ± 1.03^a^	< 0.001
FI, g	420.31 ± 4.89^a^	421.10 ± 2.31^a^	418.81 ± 2.06^a^	414.70 ± 2.27^a^	0.2970
FCR,	2.60 ± 0.04^a^	2.44 ± 0.01^b^	2.36 ± 0.02^c^	2.29 ± 0.01^c^	0.0236
SGR	5.01 ± 0.01^d^	5.16 ± 0.01^c^	5.24 ± 0.02^b^	5.28 ± 0.01^a^	0.0178
ADG, g/day	4.63 ± 0.02^d^	4.93 ± 0.03^c^	5.07 ± 0.02^b^	5.17 ± 0.03^a^	0.0024

^a, b,c, d^Means in the row with different superscript letters are significantly different at *P* < 0.05.

Initial body weight (IBW), final body weight (FBW), body weight gain (BWG), feed intake (FI), Feed conversion ratio (FCR), Average daily gain (ADG), Specific growth rate (SGR).

PSP0, PSP1, PSP2, PPS3 indicate Japanese quails feeding basal diets supplemented with 0, 10, 20 and 3 g /kg diet of phycocyanin extracted from *Spirulina Platensis* (PSP)/kg diet.

### Blood hematology

Table 4 presents the effects of dietary PSP inclusion on the blood hematology of growing Japanese quails. Feeding quails a diet fortified with 1, 2, or 3 g of PSP significantly improved the white blood cell (WBC) and eosinophil counts in a dose-dependent way (*P* < 0.01). The highest WBC and eosinophil counts were observed in the PSP3 group (*P* < 0.05). Lymphocyte counts were the lowest in the PSP3 group, while monocytes were the greatest in the PSP2 and PSP3 groups (*P* < 0.05). RBC’s values were significantly improved in a dose-dependent way by adding 1, 2 and 3 g of PSP/kg diet (*P* < 0.01). The levels of MCV did not statistically differ among all PSP groups (*P* < 0.01). All PSP diets improved PCV (except for PSP1) compared to the control diet (*P* < 0.05). Hemoglobin was the greatest in PSP3 group followed by PSP2 groups with significant effect (*P* < 0.05). Feeding quails a diet fortified with 2–3 g of PSP enhanced the levels of MCHC and MCH compared to the PSP1 and PSP0 groups (*P <* 0.01).

**Table 4:** The effects of dietary inclusion of phycocyanin extracted from *Spirulina Platensis* (0, 1, 2, and 3 g/kg diet) on the blood hematology of growing Japanese quails.

Item	Dietary addition groups				*P* values
	PSP0	PSP1	PSP2	PSP3	
WBCs (10^3^/mL)	3.35 ± 0.00^d^	3.36 ± 0.00^c^	3.38 ± 0.00^b^	3.40 ± 0.00^a^	0.0023
Eosinophil (%)	2.22 ± 0.01^d^	2.27 ± 0.01^c^	2.35 ± 0.01^b^	2.40 ± 0.01^a^	0.007
Lymphocytes (%)	90.98 ± 0.10^a^	90.48 ± 0.05^bc^	90.63 ± 0.11^b^	90.23 ± 0.05^c^	< 0.001
Monocytes (%)	2.13 ± 0.01^c^	2.31 ± 0.03^b^	2.49 ± 0.03^a^	2.47 ± 0.02^a^	< 0.001
Neutrophils (%)	2.46 ± 0.01^c^	2.53 ± 0.01^b^	2.57 ± 0.00^a^	2.59 ± 0.01^a^	< 0.001
PCV (%)	40.70 ± 0.07^c^	40.85 ± 0.03^bc^	40.95 ± 0.06^ab^	41.08 ± 0.06^a^	0.0034
RBCs (10^6^/mL)	3.74 ± 0.00^d^	3.76 ± 0.00^c^	3.77 ± 0.00^b^	3.78 ± 0.00^a^	< 0.001
Hemoglobin (g/dl)	11.28 ± 0.02^c^	11.34 ± 0.03^c^	11.51 ± 0.05^b^	11.63 ± 0.01^a^	0.007
MCV (fL)	108.72 ± 0.21	108.68 ± 0.11	108.60 ± 0.24	108.60 ± 0.08	0.0369
MCH (pg/cell)	30.14 ± 0.04^b^	30.16 ± 0.09^b^	30.51 ± 0.11^a^	30.74 ± 0.05^a^	< 0.001
MCHC (g/dL)	27.72 ± 0.04^b^	27.75 ± 0.07^b^	28.10 ± 0.14^a^	28.30 ± 0.07^a^	< 0.001

^a, b,c, d^Means in the row with different superscript letters are significantly different at *P* < 0.05.

WBCs (white blood cells), PCV (packed cell volume), RBCs (red blood cells).

PSP0, PSP1, PSP2, PPS3 indicate Japanese quails feeding basal diets supplemented with 0, 10, 20 and 3 g /kg diet of phycocyanin extracted from *Spirulina Platensis* (PSP)/kg diet.

### Blood biochemistry

The effects of phycocyanin extracted in the diet of growing Japanese quails at different levels (0, 1, 2, and 3 g/kg diet) on their blood biochemistry are shown in Table 5. The dietary addition of PSP increased total protein (TP) and ALB levels in a dose-dependent manner as the concentration increased from 1 to 3 g/kg of the diet (*P* < 0.05). The highest levels of TP and ALB were observed in Japanese quails fed a diet containing 3 g of PSP. All PSP groups had greater GLOB compared to the control diet, with the greatest increase shown in the PSP3 group (*P* < 0.05). Adding PSP to the diet of Japanese quails at concentrations of 2–3 g/kg diet significantly reduced serum ALT levels. Additionally, all PSP-supplemented groups showed a significant reduction in serum AST levels compared to the control diet (*P* < 0.05). The dietary inclusion of PSP in Japanese quail diets significantly reduced urea levels in a dose-dependent manner (*P* < 0.01). In comparison with the control diet, all PSP-added diets resulted in lower levels of creatinine in Japanese quails (*P* < 0.01). Additionally, all PSP-added diets resulted in greater levels of lysosomal activity in Japanese quails (*P* < 0.01) compared to PSP-free diets. The serum levels of TG were significantly reduced by increasing the level of PSP in a dose-dependent way. The addition of 2–3 g/kg diet significantly improved the TC compared to control diets (*P* < 0.05). Overall, supplementing diets with PSP (2–3 g/kg diet) supported the blood health markers in Japanese quails.

**Table 5:** The effects of dietary inclusion of phycocyanin extracted from *Spirulina Platensis* (0, 1, 2, and 3 g /kg diet) on the blood biochemistry of growing Japanese quails.

Item	Dietary addition groups				*P*-value
	PSP0	PSP1	PSP2	PSP3	
TP (mg/dL)	6.16 ± 0.05^d^	6.46 ± 0.02^c^	6.60 ± 0.04^b^	6.77 ± 0.04^a^	0.002
ALB (mg/dL)	3.10 ± 0.05^d^	3.29 ± 0.01^c^	3.39 ± 0.02^b^	3.50 ± 0.03^a^	0.014
GLOB (mg/dL)	3.06 ± 0.02^c^	3.17 ± 0.02^b^	3.22 ± 0.03^ab^	3.27 ± 0.02^a^	0.003
ALT (U/L)	10.30 ± 0.01^a^	10.23 ± 0.01^a^	9.98 ± 0.05^b^	9.84 ± 0.03^c^	0.001
AST(U/L)	125.11 ± 0.85^a^	120.49 ± 0.39^b^	116.96 ± 0.32^c^	116.16 ± 0.13^c^	0.001
Urea (mg/dL)	41.58 ± 0.29^a^	38.51 ± 0.28^b^	37.17 ± 0.13^c^	36.29 ± 0.21^d^	0.001
Creatinine (mg/dL)	0.55 ± 0.01^a^	0.54 ± 0.02^b^	0.53 ± 0.01^b^	0.53 ± 0.01^b^	0.001
TG (mg/dL)	61.47 ± 1.22^a^	60.11 ± 1.09^b^	58.22 ± 1.07^c^	57.23 ± 1.11^d^	0.001
TC (mg/dL)	67.34 ± 1.3^c^	68.56 ± 1.02^b^	70.88 ± 1.02^a^	71.06 ± 1.17^a^	0.001
LYZ (ng/mL)	1.52 ± 0.012^b^	1.82 ± 0.023^a^	1.98 ± 0.017^a^	2.01 ± 0.01^a^	0.001

^a, b,c, d^Means in the row with different superscript letters are significantly different at *P <* 0.05.

TP (total protein), ALB (Albumin), GLOB (Globulins), ALT (Alanine transaminase), TC (triglycerides), AST (Aspartate transaminase), TG (total cholesterol), and lysosome activity (LYZ).

PSP0, PSP1, PSP2, PPS3 = Japanese quails feeding basal diets supplemented with 0, 1, 2 and 3 g of phycocyanin extracted from *Spirulina Platensis* (PSP)/kg diet.

### Histopathological findings

#### Intestinal histology

**Fig. 1:**
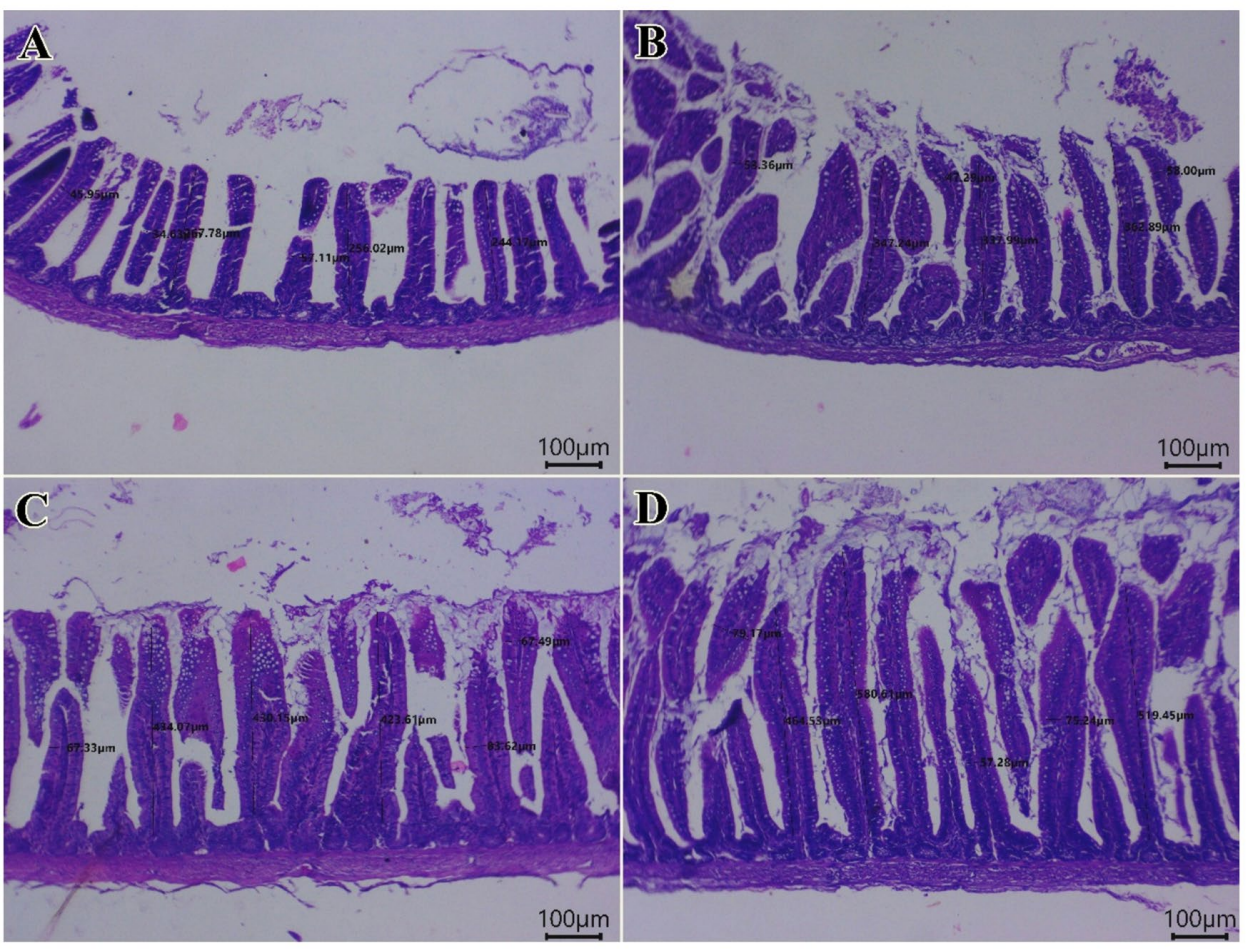
The photomicrographs of H&E-stained sections from the intestine of quails (Scale bar 100 μm) displaying: (**A**) normal configurations of enterocytes lining villous mucosa, submucosal layer, and muscular layer in the control group. Birds fed diet with 1 (**B**), 2 (**C**) and 3 (**D**) g of phycocyanin isolated from spirulina had an enhancement in morphological integrity of intestinal layers, primarily mucosal villi.

**Fig. 2:**
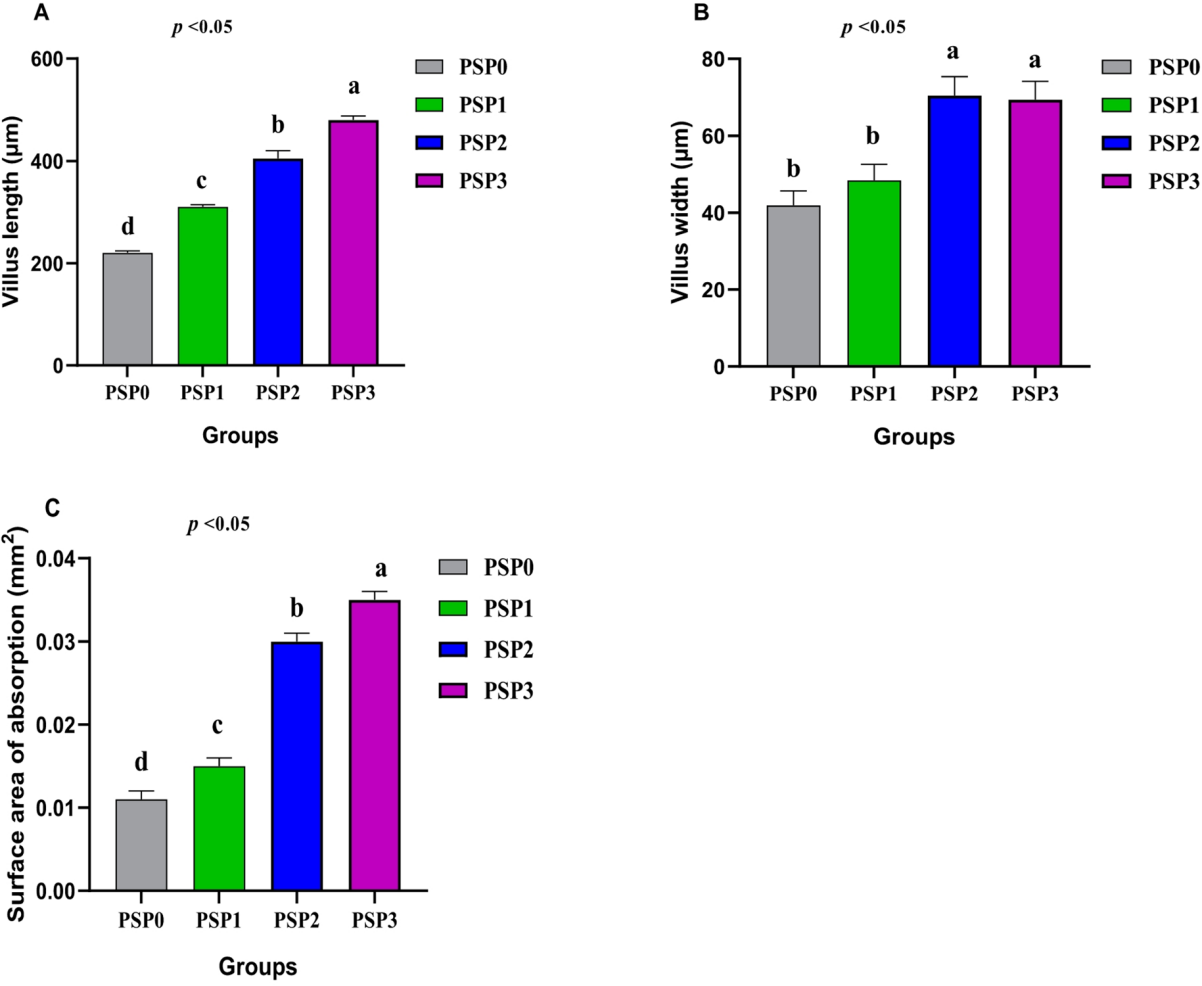
Graph showing the effect of different treatments on villus length (**A**), villus width (**B**) and surface area of absorption (**C**). The treated groups were designated as follows: control group (PSP0), supplemented with 1 g of phycocyanin isolated from *Spirulina Platensis* (PSP) per kg (PSP1), supplemented with 2 g of PSP per kg (PSP2), and supplemented with 3 g of PSP per kg (PSP3). *N* = 3.

**Fig. 3:**
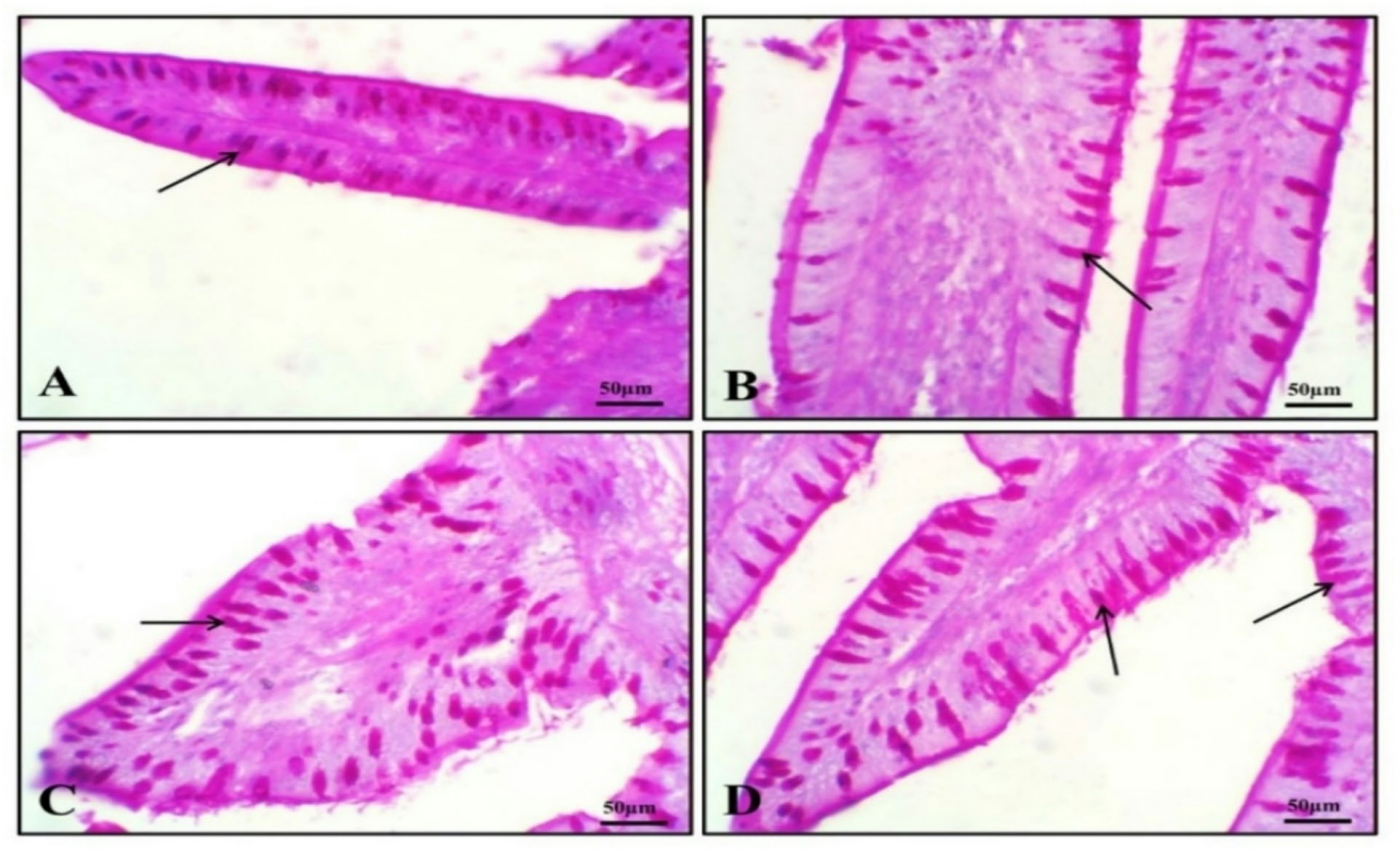
Light photomicrographs of intestinal sections from quails stained with PAS (Periodic Acid–Schiff) show PAS-positive goblet cells (arrows) in the control and experimental groups (**A**). Birds fed diet with 1 (**B**), 2 (**C**) and 3 (**D**) g of phycocyanin isolated from spirulina displayed the percentage area of intestinal goblet cells.

Histological examination of the intestinal tissues from all groups revealed a normal configuration of enterocytes, villous mucosa, submucosal layer, and muscular layer (Fig. 1). Notably, the PSP-supplemented groups at 1 (Fig. 1B), 2 (Fig. 1C) and 3 (Fig. 1D) of PSP/kg diet showed enhanced morphological integrity of the intestinal layers, particularly the mucosal villi, compared to the control group (PSP0). These morphological improvements are further supported by the histomorphometric data presented in Fig. 2, which details change in villus length (Fig. 2A), villus width (Fig. 2B) and surface area of absorption (Fig. 2C). The values of villus length (Fig. 2A) and surface area of absorption (Fig. 2C) were significantly enhanced in response to dietary increases in the levels of PSP in a dose-dependent manner (*P* < 0.05). Light photomicrographs of intestinal sections (Fig. 3) from quails stained with PAS (Periodic Acid–Schiff) show PAS-positive goblet cells (arrows) in the control and experimental groups (Fig. 3A). Birds fed diet with 1 (Fig. 3B), 2 (Fig. 3C) and 3 (Fig. 3D) g of phycocyanin isolated from spirulina displayed the percentage area of intestinal goblet cells.

#### Liver histology

**Fig. 4:**
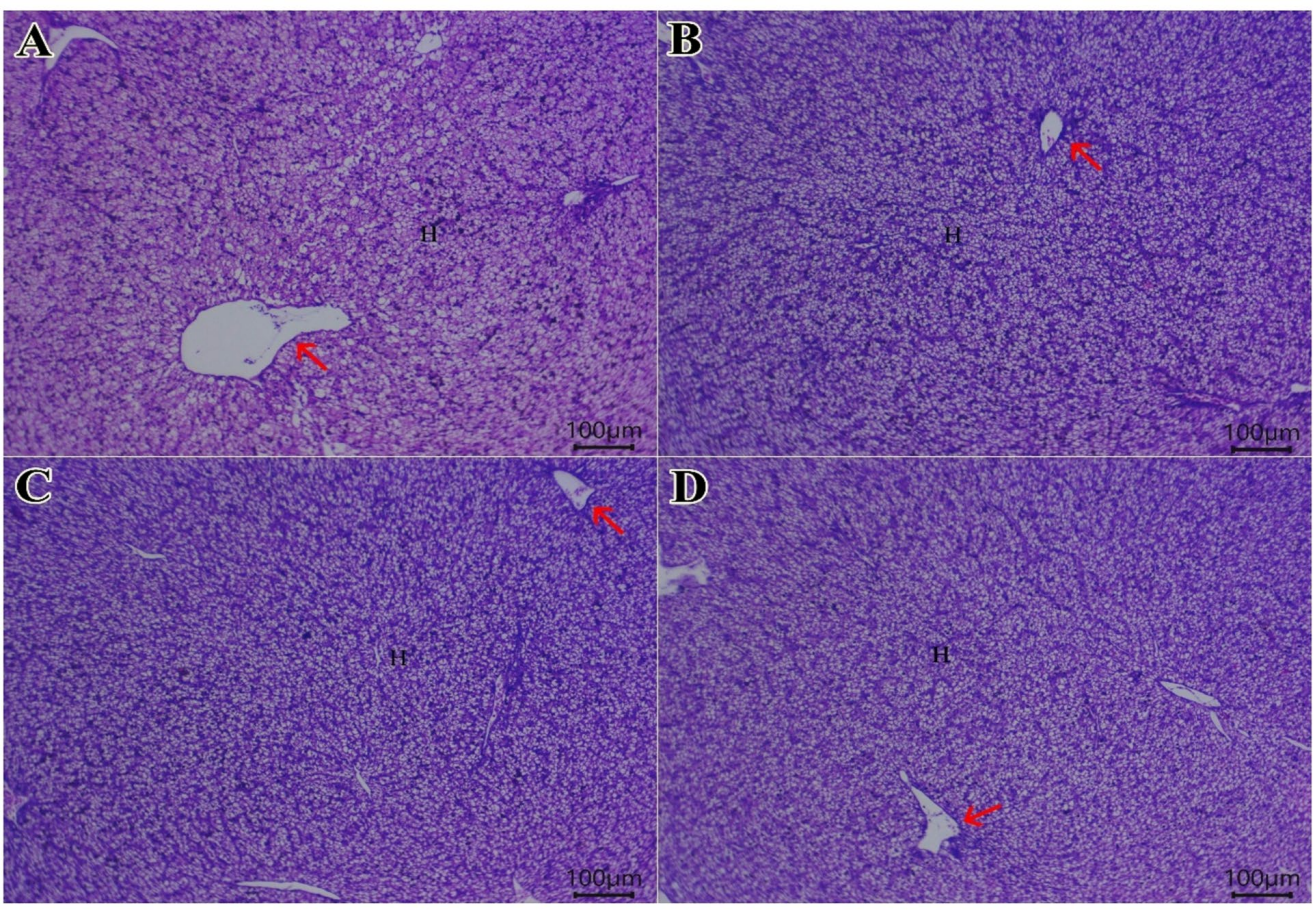
A photomicrograph of liver tissue sections stained with Hematoxylin and Eosin (H&E) shows the effects of treatment on quails. The scale bar represents 100 μm. (**A**) Control Group: Shows the normal arrangement of spherical hepatocytes (H), a central vein (red arrow), sinusoids, and portal areas. Birds fed diet with 1 (**B**), 2 (**C**) and 3 (**D**) g of phycocyanin isolated from *Spirulina Platensis* (PSP) display pathological changes including intracytoplasmic vacuolations, dilated hepatic vasculature, and active Kupffer cells.

**Fig. 5:**
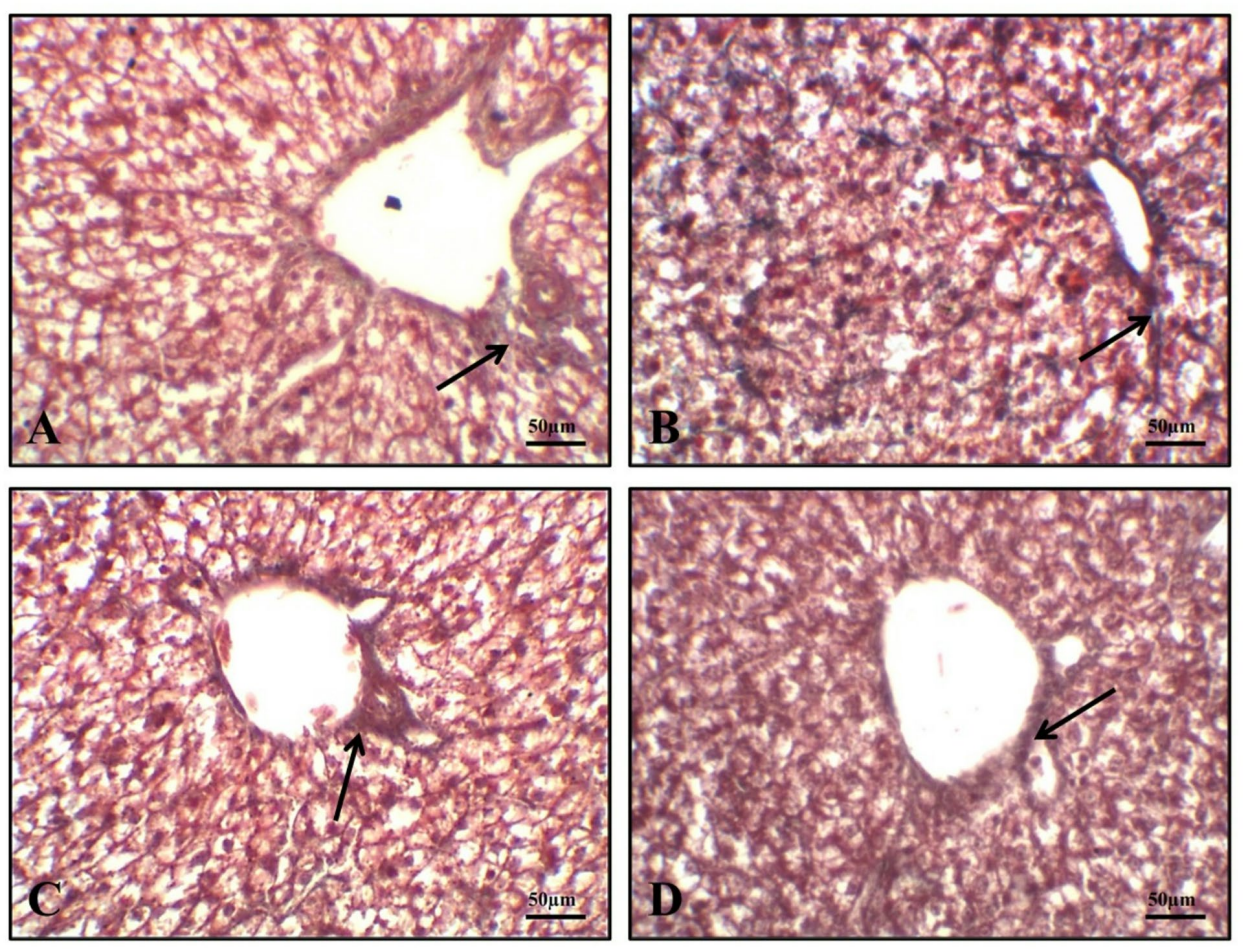
Masson’s trichrome-stained liver tissue sections show the distribution of collagen fibers (arrows) around the central vein, which is normally distributed in the control group (**A**) (Masson trichrome stain 400X). The treated groups were designated as follows: control group (PSP0), supplemented with 1 g of phycocyanin isolated from *Spirulina Platensis* (PSP) per kg (PSP1), supplemented with 2 g of PSP per kg (PSP2), and supplemented with 3 g of PSP per kg (PSP3). *N* = 3.

Examined liver sections from quails (Fig. 4) showed a normal histological arrangement of spherical hepatocytes, central veins, sinusoids, and portal areas across all groups (PSP0, PSP1, PSP2, and PSP3). However, the control group (PSP0, Fig. 4A) also exhibited pathological features, including intracytoplasmic vacuolations, dilated hepatic vasculature, and active Kupffer cells. These signs were absent or less severe in the PSP-supplemented groups. Figure 5 shows Masson’s trichrome staining for collagen. The liver tissue from all experimental groups, including the control, shows a normal distribution of collagen fibers. Consistent with histologically normal liver tissue, the staining reveals limited collagen deposition, which is primarily confined to the fibrous connective tissue surrounding the portal triads and central veins.

### Gene expression assessment

**Fig. 6:**
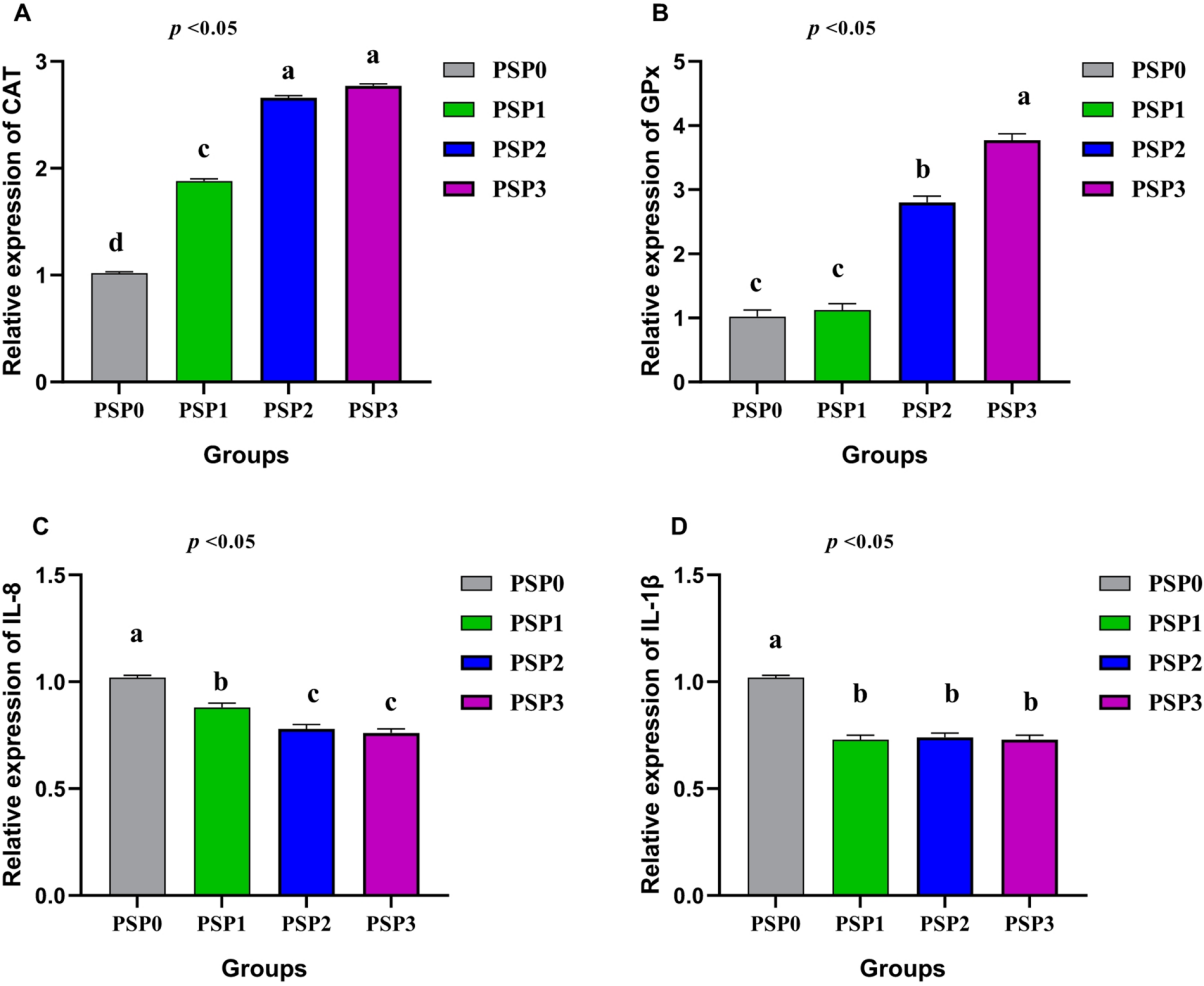
Graph showing the effect of different treatments on antioxidant genes including *CAT* (**A**), *GPx* (**B**), and proinflammatory cytokine genes such as *IL-8* (**C**) and *IL-1β* (**D**). The treated groups were designated as follows: control group (PSP0), supplemented with 1 (PSP1) or 2 (PSP2), 3 (PSP3) g of phycocyanin isolated from *Spirulina Platensis* (PSP) per kg. *N* = 3.

Figure 6 shows the effects of dietary phycocyanin on the expression of antioxidant and pro-inflammatory cytokine genes in growing Japanese quails. The inclusion levels were 1, 2, and 3 g/kg of the diet, compared to a control group. The daily addition of 2–3 g PSP significantly upgraded *CAT* (Fig. 6A) genes compared to other groups (*P* < 0.05). Additionally, birds fed 3 g of PSP had the greatest expression of *GPx* (Fig. 6B) compared to other groups, despite all PSP-supplemented groups having greater levels of GPX than the control diets (*P* < 0.05). For pro-inflammatory genes, the PSP diets led to a significant reduction in the expression of interleukin 8 (*IL-8*, Fig. 6C) and interleukin-1 beta (*IL-1β*, Fig. 6D) compared to the control diet (*P* < 0.05).

## Discussion

Due to the ban on the use of antibiotics in livestock production, nutrition scientists have intensified their research to identify more effective, eco-friendly, and sustainable sources of natural compounds as viable alternatives. Our findings demonstrate that supplementing Phycocyanin from *Spirulina platensis* significantly improved key growth indices (FBW, ADG, and FCR). Furthermore, it modulated blood metabolic parameters and positively influenced the hematological profile (RBCs, WBCs, and erythrocytes). The optimal doses of dietary PSP for improving growth and blood hematological markers in quails were found to be 2–3 g/kg diet.

Growth performance is a crucial indicator for assessing the economic viability of quail farms. In this study, quails were supplemented with phycocyanin from *Spirulina platensis* for 35 days. By the end of the trial, birds that received 3 g phycocyanin per kg of feed showed significantly improved growth performance, with higher body weight (BW), body weight gain (BWG), and a lower feed conversion ratio (FCR), compared to the control group (PSP0). These findings align with previous research by multi studies^8,39^. Several studies have shown that supplementing poultry feed with Spirulina has positive effects. Jamil et al.^40^ found that adding Spirulina to broiler feed improved growth performance, feed efficiency, and body weight. Algal supplements can slow intestinal transit and improve nutrient digestibility at levels up to 16% in the diet^41^. In Japanese quails, Abouelezz^42^ observed that *S. platensis* supplementation significantly increased body weight gain. This is attributed to the nutrient composition and functional properties of *S. platensis*, which enhances metabolic rate and developmental attributes in birds. Several studies have reported the beneficial effects of spirulina on the growth performance of quails, which is consistent with the findings of this current experiment^43–45^. The enhancement in growth may correlate with the increased secretion of digestive enzymes. A study by Abdel-Wahab et al.^43^, demonstrated that birds fed a spirulina-supplemented diet showed significantly higher levels of amylase, trypsin, and lipase compared to a control group. The improvements in growth performance may be attributed to phycocyanin’s ability to increase villus height and crypt depth in the duodenal tissue of quails^46^. This is further supported by evidence that phycocyanin can modulate the gut microbiota, promote the growth of beneficial bacteria while suppress pathogenic bacteria^46^. Under heat stress conditions, the addition of spirulina (15 g/kg) significantly improved the growth performance of broiler chickens^47^. Consistent with our results, it has been shown that in ovo injection of spirulina (at a dose of 1.5–3.5 mg/egg) enhanced the growth performance, FI, and FCR)of broilers compared to an untreated control group^48^. In contrast, the growth of chickens was not affected by the addition of 0.25–1 g of phycocyanin to their diet, according to research by Amer et al.^49^.

The inclusion of phycocyanin in broiler diets at concentrations of 0.25–1 g/kg for 35 days did not affect carcass yield, with results being comparable to the control basal diet. The addition of spirulina (at a concentration of 10–15 g/kg of feed) to the diet of broilers under heat stress significantly improved carcass traits^47^. This improvement suggests that spirulina supplementation can help mitigate the negative effects of heat stress on meat yield and quality^47^. In contrast, dietary supplementation with a mixture of Dunaliella and Arthrospira platensis (at a concentration of 0.5–1 g/kg) did not significantly affect the carcass traits of quails^45^.

Blood hematology is the initial step for a comprehensive health assessment, serving as a diagnostic tool to evaluate an organism’s nutritional status, overall health, and the presence of infections or other disorders. Our investigation found that supplementing PSP improved key hematological parameters, including hemoglobin, RBC, and WBC counts. Chicks fed a diet with 0.25 g of phycocyanin showed improved blood hematology, with higher counts of red blood cells, white blood cells, and heterophils, as well as increased hemoglobin content, compared to the control group and those fed 0.5 g and 1 g (*P* < 0.05)^49^. The observed increase in blood hemoglobin is likely a direct result of the extract’s rich mineral content and its positive effect on intestinal integrity and permeability^44^. This aligns with similar findings by Alwaleed et al.^50^ who reported that adding *Spirulina* powder to broiler diets also raised hemoglobin levels.

The inclusion of phycocyanin in broiler diets at concentrations of 0.25–1 g/kg for 35 days did not affect AST, ALT, creatinine, or uric acid with results being comparable to the control basal diet^49^. We also assessed several clinical serum biochemicals, such as total protein, globulin, the albumin/globulin ratio, and markers for liver and kidney function. Feeding Japanese quails with algal derivatives (phycocyanin and fucoidan) in their drinking water at concentrations of 20 or 40 mg/L significantly (*P* < 0.01) reduced their levels of total cholesterol, triglycerides, and creatinine^51^. Algal derivatives (phycocyanin and fucoidan) in their drinking water at concentrations of 20 or 40 mg/L significantly reduced Yolk cholesterol in Japanese quails^46^. Feeding stressed Japanese quails a diet supplemented with spirulina at 10–15 g/kg significantly improved blood protein levels and reduced the activity of liver enzymes (AST and ALT). This was also accompanied by a decrease in lipid profile and uric acid levels^47^. The addition of spirulina to the diet of growing Japanese quails at a concentration of 4.5% significantly improved their liver function and reduced their total lipid profile^43^. Consistent with our findings, dietary supplementation with a mixture of Dunaliella and *Arthrospira platensis* (at a concentration of 0.5–1 g/kg) significantly improved blood protein levels, reduced hepatic enzyme activity, and decreased blood lipid levels^45^.This outcome is consistent with previous research demonstrating the beneficial effects of spirulina on the health and growth of quails. According to Hajati and Zaghari^26^*Spirulina platensis* lowers cholesterol levels. Our findings indicate that *Spirulina* supplementation in quail diets significantly lowered total cholesterol while increasing triglyceride levels compared to the control group. This aligns with previous research that reported a significant reduction in blood serum cholesterol levels from *Spirulina* supplementation (*P* < 0.05)^52^. We also note that some studies contradict these conclusions^53^. Furthermore, supplementation with *S. platensis* has been shown to improve other blood parameters. Our findings are consistent with Sugiharto et al.^35^, who reported that *Spirulina platensis* additions to broiler diets produced similar results. Other studies have also shown that *Spirulina* supplementation can significantly lower cholesterol and triglyceride levels in broilers (*P* < 0.05)^54^. In our current study, we observed that quails fed a diet supplemented with *S. platensis* had significantly lower levels of both liver enzymes (ALT, AST) and kidney function indicators (*P* < 0.05) compared to the control group. This supports previous research demonstrating that *S. platensis* supplementation improves renal and liver function in quails^40^. The hepatoprotective and nephroprotective effects of *Spirulina* have been well documented. However, it is important to note that some findings are contradictory, as Choi et al.^55^ found that algal byproducts increased levels of blood albumin, total cholesterol, and triglycerides while having no effect on globulin, AST, or BUN in laying hens.

Antioxidant enzymes (CAT, GPX and SOD) of quails fed diet with 4.5% spirulina were greater than quails of control diet^1,43^. Algal derivatives (phycocyanin and fucoidan) in their drinking water at concentrations of 20 or 40 mg/L significantly reduced Yolk MDA in Japanese quails^46^. Additionally, stressed quails fed diets supplemented with spirulina at a concentration of 10–15 g/kg showed a significant decrease in MDA and the inflammatory cytokines IL-β1 and TNF-α^47^.

The internal and immune-related organs was not affected by the addition of 0.25–1 g of phycocyanin to their diet, according to research by Amer et al.^49^. In ovo administration of spirulina (1.5–3.5 mg/egg) to broilers upregulated the expression of *HSP70* and *GPx* genes while downregulating the expression of interferon-gamma (IFN-γ)^48^. Adding 0.25–1 g of phycocyanin significantly improved the lysozyme, interleukin-10, and complements 3 serum levels in broiler^49^. The dietary inclusion of a mixture of Dunaliella and *Arthrospira platensis* (at a concentration of 0.5–1 g/kg) resulted in significantly higher levels of complement 3 and lysozyme in quails^45^. This suggests an improvement in the birds’ innate immune response. Dietary supplementation with *Spirulina platensis* (4.5%) significantly modulated the intestinal microbiota, leading to a significant increase in the population of Lactobacillus sp. while substantially decreasing the populations of *Escherichia coli* and Salmonella^1^.

Phycocyanin from *Spirulina platensis* is known to repair damaged liver cells and protect renal tissues from toxicity by eliminating free radicals^56,57^. Our histological analysis of the intestine and liver demonstrated that birds treated with the *S. platensis* extract showed significant, dose-dependent improvements in intestinal structure compared to untreated controls. This finding likely explains the enhanced production metrics, as improved intestinal villi histology leads to better nutrient absorption, which is reflected in the birds’ higher FBW and lower FCR. Furthermore, all treatment groups had normal hepatocyte structures, indicating that varying doses of the *S. platensis* extract have no harmful effects on liver morphology. Our findings were consistent with those of Alghamdi et al.^8^ who found that the histological analysis of the intestine and liver indicated a significant improvement in Japanese quail diets treated with *S. platensis*.

Research has shown that *Spirulina* has protective effects on both the liver and intestines. Abdulla et al.^58^ reported that *Spirulina* powder reduces histological changes in rats with induced liver injury, and Morsy et al.^59^ found that *S. platensis* extract protects the intestines of rats from induced ulcerative colitis. Goblet cells are specialized epithelial cells found in the intestinal lining. They secrete mucus, which forms a protective barrier to maintain the integrity of the intestinal mucosa and prevent pathogen invasion and inflammation^56,60^. Recent research has redefined their function, highlighting their active role in mucosal immunity and intestinal homeostasis^61^. Goblet cells are central to maintaining gut homeostasis. Through mucus and antimicrobial peptide secretion, they create a protective role that supports beneficial microbes while defending against pathogens^62^.

The histological examination of liver tissues in the present study revealed a normal distribution of collagen fibers in both the control and phycocyanin-treated groups. Masson’s trichrome staining exhibited minimal collagen deposition, primarily localized around the portal triads and central veins, which is consistent with the architecture of healthy liver tissue. This suggests that Spirulina’s bioactive components, such as phycocyanin and polysaccharides, have been shown to scavenge free radicals and do not induce fibrotic changes, and may help preserve normal extracellular matrix organization^56^. This finding is consistent with the studies of Coué et al.^63^, who reported that oral supplementation with Spirulina liquid extract protects mice against hepatic fibrosis, inflammation, oxidative stress, and insulin resistance induced by a Western diet.

Phycocyanin (PSP) act as antioxidants, protecting cells from oxidative damage^64^. Oxidative stress can impair growth by destroying cellular components, but antioxidant genes like CAT and GPx can mitigate this by lowering oxidative stress levels. Our findings show that PSP supplementation not only improved antioxidant status but also enhanced cellular immunity, as evidenced by increased *expression* of *IL-8* and *IL-1β* genes. Specifically, the PSP3 group demonstrated the highest concentrations of these cytokines, indicating a strong positive effect on both antioxidant status and cellular immunity. Dietary antioxidants play a crucial role in neutralizing reactive oxygen species, thereby inhibiting inflammatory reactions^65^. Overall, PSP supplementation improved the birds’ antioxidant status by upregulating *GPx* and *CAT* gene *expression*. Simultaneously, it mitigated the inflammatory response by downregulating the expression of IL-8 and IL-1β genes. The use of predicted gene sequences was one of the primary limitations of this study. However, these sequences were utilized as they represented the most comprehensive genomic data available for this species in public databases, and their reliability was further supported by high homology scores during BLAST analysis. Furthermore, we recognize the importance of conducting an economic feasibility analysis for practical application. We intend to incorporate a detailed cost-benefit analysis in our future research.

## Conclusion

Dietary supplementation with *Spirulina platensis*-derived phycocyanin (PSP) significantly enhances growth performance, feed efficiency, and organ histomorphology in Japanese quails, with optimal results observed at 2–3 g/kg diet. These improvements are linked to the upregulation of antioxidant and immune-related genes. Further investigations are required to isolate the specific bioactive compounds responsible for these immunomodulatory and antioxidant effects. Elucidating the precise molecular interactions between these microalgae-derived compounds and avian physiology will be crucial for developing targeted nutritional strategies to optimize poultry health and productivity.

## Data Availability

All data generated or analyzed during this study are included in this published article.
